# β-Glucans as Dietary Supplement to Improve Locomotion and Mitochondrial Respiration in a Model of Duchenne Muscular Dystrophy

**DOI:** 10.3390/nu13051619

**Published:** 2021-05-12

**Authors:** Letizia Brogi, Maria Marchese, Alessandro Cellerino, Rosario Licitra, Valentina Naef, Serena Mero, Carlo Bibbiani, Baldassare Fronte

**Affiliations:** 1 BIO@SNS, Scuola Normale Superiore, via Moruzzi 1, 56126 Pisa, Italy; letizia.brogi@sns.it (L.B.); alessandro.cellerino@sns.it (A.C.); 2 Molecular Medicine & Neurobiology-ZebraLab, IRCCS Fondazione Stella Maris, 56128 Pisa, Italy; maria.marchese@fsm.unipi.it (M.M.); rosario.licitra@fsm.unipi.it (R.L.); Valentina.naef@fsm.unipi.it (V.N.); s.mero27@gmail.com (S.M.); 3 Leibniz Institute on Aging, Fritz Lipmann Institute, 07745 Jena, Germany; 4 Dipartimento di Scienze Veterinarie, Università di Pisa, 56124 Pisa, Italy; carlo.bibbiani@unipi.it

**Keywords:** β-glucans, DMD, locomotion, mitochondrial function, zebrafish

## Abstract

Duchenne muscular dystrophy (DMD) is a severe X-linked neuromuscular childhood disorder that causes progressive muscle weakness and degeneration. A lack of dystrophin in DMD leads to inflammatory response, autophagic dysregulation, and oxidative stress in skeletal muscle fibers that play a key role in the progression of the pathology. β-glucans can modulate immune function by modifying the phagocytic activity of immunocompetent cells, notably macrophages. Mitochondrial function is also involved in an important mechanism of the innate and adaptive immune responses, owing to high need for energy of immune cells. In the present study, the effects of 1,3-1,6 β-glucans on five-day-old non-dystrophic and dystrophic (*sapje*) zebrafish larvae were investigated. The effects of the sonication of β-glucans and the dechorionation of embryos were also evaluated. The results showed that the incidence of dystrophic phenotypes was reduced when dystrophic embryos were exposed to 2 and 4 mg L^−1^ of 1,3-1,6 β-glucans. Moreover, when the dystrophic larvae underwent 8 mg L^−1^ treatment, an improvement of the locomotor performances and mitochondrial respiration were observed. In conclusion, the observed results demonstrated that 1,3-1,6 β-glucans improve locomotor performances and mitochondrial function in dystrophic zebrafish. Therefore, for ameliorating their life quality, 1,3-1,6 β-glucans look like a promising diet supplement for DMD patients, even though further investigations are required.

## 1. Introduction

Duchenne muscular dystrophy (DMD) is characterized by a progressive muscle degeneration leading to complete immobility and death. The disease is caused by mutations in DMD (encoding dystrophin) that abolish the production of dystrophin in muscle [1,2,3]. Loss of dystrophin initiates a complex series of pathophysiological changes that muscle weakness, causes contractures, joint deformities, and locomotor disorders [4,5]. To date, there is still no cure for DMD, and even though steroid therapy and support strategies have improved patient survival and life quality, they are accompanied by important short- and long-term negative side effects [6,7,8]. Recently, research on DMD therapies, has focused on possible gene-based strategies, and a few of these are now reaching the phase II/III clinical trial stage, such as the upregulation of utrophin, the enhancement of muscle regeneration, and virus-mediated mini-dystrophin gene therapy [9]. Meanwhile, two therapies have been approved by international regulatory agencies: one is based on stop codon readthrough, whereas the other uses an exon-skipping strategy to restore production of internally deleted, yet efficient, dystrophin [10]. However, approved drugs have shown only partial success in DMD and, therefore, remain suitable for relatively few patients, i.e., those with specific mutations. Moreover, a new approach was proposed for cell-based therapy for Duchenne muscular dystrophy [11,12], which is based on the use of cells called muscle side population (SP) cells to deliver genes, such as the human micro-dystrophin, showing their capability in recapitulating the myogenic lineage. Pharmacological corticosteroid therapy and other supportive strategies are the standard of care in DMD, which have improved survival and health-related quality of life in this setting. Nevertheless, the clinical efficacy and short-term benefits and significant adverse effects associated with long term-use of steroid is established. Alternative non-steroidal therapeutics that can be rapidly translated into a clinical setting should also be investigated for the treatment of DMD patients [13]. Empirical experiences reported by DMD patients suggest that diets supplemented with bioactive compounds may improve muscle strength [14]. Messina et al. observed that adequate nutrition exerts anti-inflammatory effects and delays the onset of muscle atrophy in both DMD patients and validated mouse (*mdx*) model [15]. Moreover, in *mdx* mice, long-term administration of a low-protein diet led to a significant reduction in inflammation and muscle function improvement [16]. The reduction in muscle strength in DMD patients is probably caused by the accumulation of damaged cell organelles resulting in increased oxidative stress [16]. Further, oxidative stress reduces muscle strength by worsening mitochondrial respiration [17]. Indeed, an important feature of DMD is a compromised bioenergetic status caused by a reduced capacity of OXPHOS in dystrophic mitochondria, as it has been constantly reported in the literature [18]. For these reasons, many antioxidant compounds such as Coenzyme Q10, resveratrol, melatonin, gingerol, and preparations of traditional Chinese medicine have been tested to counteract the negative effects of DMD [19,20,21]. The antioxidant action of resveratrol, natural polyphenol, significantly decreased the muscular reactive oxygen species (ROS) levels and ameliorated the pathology of *mdx* mice [20]. Medical–scientific interest in “functional” foods as modifiers of the biological response (BRM) has recently increased and notably for mushroom derivatives and their medical properties [22,23]. β-glucans are a group of β-D-glucose polysaccharides naturally occurring in the cell walls of cereals, bacteria, yeast, and fungi. β-glucans are a heterogeneous group of glucose polymers, consisting of a backbone of β-(1,3) linked β-D-glucopyranosyl units with β-(1,6) linked side chains that vary in distribution and length [24]. β-glucans derived from different sources have differences in their structure but are usually termed by the common name “β-glucans” [25]. Oat and barley β-glucans are linear with β (1,4) and (1,3) linkages. Mushrooms β-glucans have short β-(1,6) linked branches from β-(1,3) backbone. Yeast β-glucans have β (1,6) branches further with additional β (1,3) regions. These structural differences can determine extraction difficulties as well as bioactivity differences. Glucans characterized by larger molecular weight stimulate phagocytes, cytotoxic, and antimicrobial activities of leukocytes, together with the production of reactive oxygen species (ROS) [24]. Moreover, it has been shown that insoluble 1,3-1,6 β-glucans possess greater biological activity than the soluble (1,3-1,4) counterparts [26]. They are worldwide very well-known and already used for their strong and documented immunostimulant activity [24,25,27,28,29,30]. β-glucans exert immune modulatory activity by increasing phagocytosis and proliferation of phagocytes, granulocytes, monocytes, and macrophages [22,31]. Recently, they have been proposed as powerful immunomodulation agents for both human and zebrafish larvae [27,32]. Recently, it has been demonstrated that β-glucans reduce the expression levels of proinflammatory cytokines such as Tumor necrosis factor-α (TNF-alpha) [33], which is a cytokine that is up-regulated in DMD patients [34], contributing to chronic inflammation during disease progression [35].

When β-glucans are recognized by pattern recognition receptors (PRRs) expressed on macrophages membrane, they act as pathogen associated molecular patterns (PAMPs) inducing an enhanced immune-response [36]. The antioxidant property of β-glucans is probably due to their polymeric structure, which provides free radical removal capabilities, and they showed antioxidant properties in internal organs in vivo [37,38,39]. This suggests that β-glucans act as an antioxidant and protects the body from the adverse effects of reactive oxygen species (ROS), whose main source of their production are the mitochondria, which are also considered as targets of oxidative damage. In fact, mitochondrial ROS could cause damage to mitochondrial structures and function, due to their very high reactivity. Thus, it is possible that the enhancement of phagocytosis promoted by the dietary β-glucans [22,31] also determines the regulation of oxidative stress in DMD. To this regard, it has already demonstrated that β-glucans have a positive effect in several diseases characterized by an autophagy impairment and oxidative stress, such as cancer, Alzheimer’s, AIDS, cardiovascular disorders, diabetes, and others [28,37,40,41,42]. Yet, according to Shaki and Pourahmad, β-glucans attenuated mitochondrial reactive oxygen species (ROS) formation, lipid peroxidation and glutathione oxidation. β-glucan also prevented the loss of mitochondrial membrane potential (MMP) and mitochondrial swelling, concluding that β-glucans may prevent mitochondrial outer membrane damage and cytochrome c release from mitochondria [43]. Thanks to their immunomodulatory [30], antioxidative properties [44,45,46,47] and possible involvement in the regulation of autophagy [36], 1,3-1,6 β-glucans might be effective in muscular dystrophies also. Furthermore, since β-glucans are classified as a feed/food component (or ingredient) of “natural” origin and not a pharmaceutical compound, interesting perspectives arise for the research field and their future applications [25].

The use of zebrafish (*Danio rerio*) *sapje* strain as a model of DMD, is already known [48,49,50]. Similar to mammals for muscle structure and development, early-stage zebrafish embryos also display a high presence of homologous genes related to the muscle structure [50,51,52,53]. Moreover, at 72 hpf, larvae lacking “utrophin” protein show early muscle lesions such as a disorganized muscle structure, which is typical of DMD [53]. Differently than mammals, the early muscle degeneration in zebrafish *sapje* enables to quickly evaluate the disease evolution and the effect of possible treatments. Indeed, zebrafish *sapje* became an extremely valuable tool to evaluate the effectiveness of new molecules, compounds, and drug [49]. To this regard, it is crucial considering that when testing molecules characterized by a high molecular weight on zebrafish eggs, the chorion may represent a barrier that unable the tested molecules to reach the embryo [54]. In these cases, the sonication may reduce the size of the molecule and/or their agglomeration degree, with that enabling the chorion passage. On the other hand, the sonication itself may also cause a modification of the molecule’s bioactivity, in turn affecting the effectiveness of the treatments [55,56,57].

To identify new dietary opportunity for improving DMD patient’s well-being, the main aim of the present study was to investigate the effects of 1,3-1,6 β-glucans extracted from *Saccharomyces cerevisiae* cell wall, using *sapje* zebrafish mutant embryos as DMD animal model. To this purpose, the most effective method for performing the treatment on zebrafish eggs was also studied.

## 2. Materials and Methods

### 2.1. Ethics Statement

The study has been performed in accordance with the European Union (EU) Directive 2010/63/EU for animal experiments and according to the procedures established by the Institutional Animal Care and Use Committee (IACUC) of the University of Pisa.

### 2.2. Setting and Study Design

The present study concerned the effect of 1,3-1,6 β-glucans on a DMD animal model (zebrafish *sapje*). It was carried-out at the “zebrafish facility” of the Department of Veterinary Sciences of the University of Pisa, in collaboration with IRCCS Fondazione Stella Maris (Scientific Hospitalization and Care Institute), Pisa (Italy).

### 2.3. Zebrafish Maintenance and Embryos Production

The study was carried out using wild type strain AB and *sapje* zebrafish line [48]. According to Lawrence et al. [58], the adult zebrafish (*sapje* and wild type) were maintained at 28 °C in a water recirculating system, under a 12 h light: dark photoperiod. Mating was performed using a single couple tank 0.8 L capacity and eggs were collected, washed with egg water, and incubated at 28 °C until hatching.

### 2.4. Treatments

At 24 h post fertilization (hpf), viable eggs were distributed in 5.5 cm diameter Petri dishes containing 10 mL of egg water each. The treatments consisted of 3–4 replicates and 20–30 eggs per replicates (according to egg availability). Before to perform the treatment, a 20 mg mL^−1^ stock suspension of 1,3-1,6 β-glucans extracted from *Saccharomyces cerevisiae* cell wall (MacroGard^®^, Biorigin^©^, São Paulo, Brazil) was prepared and then added to the Petri dishes to obtain the following β-glucans concentrations: 0 (untreated), 2, 4 and 8 mg L^−1^. Hence, each β-glucans concentration was tested combining the use of sonicated (S) or non-sonicated (NS) β-glucans with dechorionated (D) and non-dechorionated (ND) embryos. In conclusion, each β-glucans concentration was tested with: (1) non- dechorionated eggs and non-sonicated β-glucans (NDNS); (2) dechorionated embryos and non-sonicated β-glucans (DNS); (3) non-dechorionated eggs and sonicated β-glucans (NDS); (4) dechorionated embryos and sonicated β-glucans (DS). Finally, the embryos exposure to 1,3-1,6 β-glucans lasted from 24 hpf to 120 hpf, when a birefringence test was performed (see below).

### 2.5. Dechorionation Procedure

At 24 hpf, butches of 40–50 embryos were incubated for 30 min in 1 mg mL^−1^ Pronase^®^ (Sigma-Aldrich, Steinheim, Germany) solution. Afterwards, embryos were rinsed three times for 3 min with egg water [54].

### 2.6. Sonication Procedure

Twenty mL aliquots of the stock suspension were sonicated using a 3 mm probe and a “Sanyo Gallenkamp MSE Soniprep 150 Ultrasonic Disintegrator” sonicator. The sonication process lasted 2 min and consisted of four 30 s on-off cycles. During sonication, the suspension was kept on ice to prevent possible excessive overheating.

### 2.7. Birefringence Test

**Figure 1:**
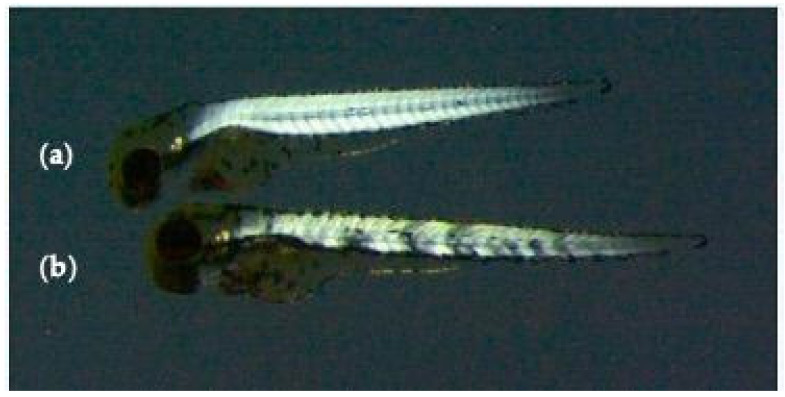
Birefringence of non-dystrophic (**a**) and dystrophic (**b**) larva.

At 96 hpf, the birefringence test was performed on *sapje* larvae. According to the structure of the muscle tissues, the larvae were classified into phenotypically dystrophic (homozygous *sapje*) and non-dystrophic (heterozygous *sapje*) larvae. The phenotypic differences between dystrophic and non-dystrophic muscle are shown in Figure 1. To this purpose, anesthetized larvae were placed between two overlapped polarized lenses and observing the muscle structure under a Leica M205FA stereomicroscope (Leica, Germany). A variable magnification ranging between 7.5× and 60× was used.

### 2.8. DanioVision

DanioVision^®^ (Noldus^©^, Wageningen, Wageningen, The Netherlands) is a complete activity monitoring system for zebrafish larvae interfaced with a specific video tracking software (EthoVision XT^®^, Noldus^©^, Wageningen, The Netherlands) that allowed to measure the following parameters: (a) distance travelled (mm); (b) velocity (mm/s); (c) Movement Cumulative Time (MCT; s). MCT is the total time spent moving by larvae over the duration of the experiment. To this purpose, at 120 hpf the treated larvae were transferred into 96 multiwell plates containing 100 μL egg water per well. Hence, one plate per time was placed in the DanioVision^®^ device and larval activity monitored for 30 min. Finally, EthoVision XT^®^ software produced a spreadsheet data set, suitable for subsequent statistical analysis.

### 2.9. Quantitative Reverse Transcription Polymerase Chain Reaction (qRT-PCR)

Total RNA was extracted from larvae at 120 hpf using Quick RNA miniprep (Zymo Research, Irvine, USA) according to the manufacturer’s instruction. cDNA and qRT-PCR were performed as described in [21]. Relative expression levels of the gene were calculated using the 2^−ΔΔCt^ method [59]. The results obtained were normalized to the expression of the housekeeping gene, *β-actin* (ENSDARG00000037746). The sequences of the primers used are listed in Supplementary Table S1. The mean of the controls (non-dystrophic larvae) was set equal to one. Each assay was done in triplicate and 31 larvae per group were analysed.

### 2.10. Mitochondrial Respiratory Analysis

Mitochondrial respiration was performed using the XF24 extracellular flow analyzer (Seahorse Bioscience, Billerica, MA, USA). The dual analyte sensor cartridges were immersed in the XF calibrator solution (Seahorse Bioscience, Billerica, MA, USA) in 24 cell culture microplates (Seahorse Bioscience, Billerica, MA, USA) overnight at 28 °C to hydrate. About 30 min before the trial period, the appropriate injection cartridges were refilled on the sensor cartridge. For this experiment was used oligomycin at a concentration of 25 µM, FCCP at a concentration of 5 µM and Rotenone plus antimycin A at a concentration of 5 µM. The 120 hpf live larvae were staged and placed in 20 of 24 wells on a microplate on the islet, placing one larva per well. The islet plate acquisition screens were placed on the measurement area to hold the larvae in place. Four wells were left empty as a control, following the already published protocols on the use of the Seahorse analyser on zebrafish larvae [60,61,62]. Each well was filled with 500 µL of egg water (pH 7.4). For the measurement of stable basal respiration, 4 repetitions of each mixing cycle of 15′′, 1′ waiting and 2′ of measurement were performed. At the end of the fourth cycle, oligomycin (an ATP synthase inhibitor) was added by performing 5 repetitions of the 15′′ mix cycle, 1.30′ wait, 3′ measurement. Subsequently, FCCP (mitochondrial protonophore/decoupling) was added by performing 4 repetitions of the measurement cycle 15′′ mix, 1.30′ wait, 3′ measurement. Finally, rotenone and antimycin A (respectively complex I and III inhibitor) were added by performing 8 repetitions of the 5′′ mixing cycle, expected from 1.30′, 3′ measurement. Using this analysis, the following parameters were measured: basal respiration, consumption of non-mitochondrial oxygen, ATP production, maximal respiration, loss of protons, spare respiratory capacity, and coupling efficiency.

### 2.11. Statistical Analysis

The data related to distance travelled, movement cumulative time, and qRT-PCR data were analysed by ANOVA. The velocity data were analysed by ANCOVA considering the distance travelled per unit of time. The observed means were compared by Tukey–Kramer HSD (honestly significant difference) test. The incidence of dystrophic and non-dystrophic phenotypes was analysed by Chi-squared test. Mitochondrial respiration data were analysed by Bonferroni-corrected Mann–Whitney U-test non-parametric. Differences between treatments were considered significant for *p* ≤ 0.05 (*), *p* ≤ 0.01 (**), *p* ≤ 0.001 (***) and *p* ≤ 0.00001 (****). Not significant differences have been shown with “ns”.

## 3. Results

### 3.1. Locomotor Performances of the Dystrophic and Non-Dystrophic Phenotypes

**Figure 2:**
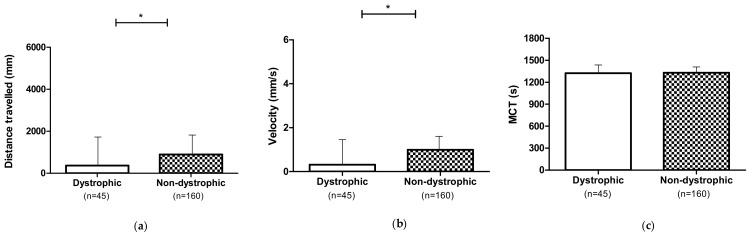
Locomotor performances of untreated (0 mg L^−1^) dystrophic and non-dystrophic larvae: (**a**) Distance travelled; (**b**) Velocity; (**c**) MCT. Data are given as mean and standard error. (* *p* ≤ 0.05).

The locomotor analysis was performed on untreated (0 mg L^−1^) dystrophic (homozygotes) and non-dystrophic larvae (heterozygotes) *sapje* mutants. Dystrophic mutants showed a reduced spontaneous locomotor activity compared to non-dystrophic subjects. In detail, dystrophic individuals travelled a shorter distance (*p* ≤ 0.05) at lower velocity (*p* ≤ 0.05), while no differences (*p* > 0.05) were observed for MCT (Figure 2).

### 3.2. Evaluation of the Best Method for Administration of 1,3-1,6 β-Glucans

Embryo dechorionation (D) and β-glucans sonication (S) might affect the effectiveness of the exposure to 1,3-1,6 β-glucans. Their effect was investigated by evaluating the frequency of phenotypically dystrophic and non-dystrophic embryos and the locomotor performances, such as distance travelled (mm), velocity (mm/s) and MCT (s).

#### 3.2.1. Effect of Embryo Dechorionation (D)

**Figure 3:**
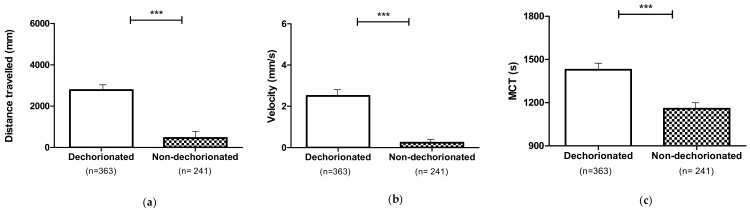
Locomotor performances of dechorionated and non-dechorionated larvae (dystrophic and non-dystrophic): (**a**) Distance travelled; (**b**) Velocity; (**c**) MCT. Data are given as mean and standard error. (*** *p* ≤ 0.001).

The analysis of the locomotor performances of dechorionated larvae (untreated or 0 mg L^−1^ and treated or 2, 4, and 8 mg L^−1^) of the two different phenotypes (dystrophic and non-dystrophic) showed an increased distance travelled (*p* ≤ 0.001), velocity (*p* ≤ 0.001) and MCT (*p* ≤ 0.001) in comparison to the non-dechorionated ones (Figure 3).

#### 3.2.2. Effect of 1,3-1,6 β-Glucans Sonication (S)

**Figure 4:**
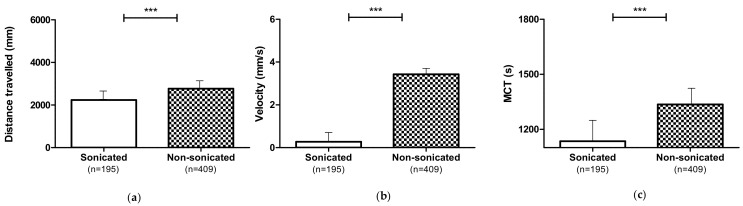
Locomotor performances of larvae (dystrophic and non-dystrophic) treated with non-sonicated or sonicated 1,3-1,6 β-glucans at 2, 4, and 8 mg L^−1^: (**a**) Distance travelled; (**b**) Velocity; (**c**) MCT. Data are given as mean and standard error. (*** *p* ≤ 0.001).

The larvae exposure to sonicated 1,3-1,6 β-glucans (2, 4 and 8 mg L^−1^), dystrophic and non-dystrophic, showed a negative effect on the locomotion performances (Figure 4), inducing a reduction of distance travelled (*p* ≤ 0.001), velocity (*p* ≤ 0.001), and MCT (*p* ≤ 0.001).

#### 3.2.3. Combined Effect of Embryo Dechorionation and Sonication

**Figure 5:**
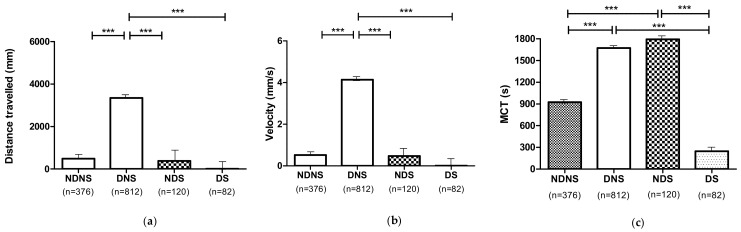
Locomotor performances of the 4 treatments obtained by combining non-dechorionated and dechorionated embryos with sonicated and non-sonicated 1,3-1,6 β-glucans (NDNS, DNS, NDS and DS); all larvae, untreated (0 mg L^−1^) and treated at 2, 4 and 8 mg L^−1^ of 1,3-1,6 β-glucans, were used. (**a**) Distance travelled; (**b**) Velocity; (**c**) MCT. Data are given as mean and standard error. (*** *p* ≤ 0.001).

The locomotor performances of all larvae (dystrophic and non-dystrophic, untreated (0 mg L^1^) and treated with 2, 4 and 8 mg L^−1^ 1,3-1,6 β-glucans) were also evaluated considering the combination of embryo dechorionation and β-glucans sonication (NDNS, DNS, NDS and DS). Dechorionated embryos treated (2, 4, and 8 mg L^−1^) with non-sonicated β-glucans (DNS) improved the larvae locomotor performances. In detail, DNS showed greater distance travelled (*p* ≤ 0.001) and velocity (*p* ≤ 0.001) than the other groups (DS, NDS and NDNS). Furthermore, both DNS and NDS showed a higher MCT than the DS and NDNS groups (*p* ≤ 0.001). Detailed differences between treatments are shown in Figure 5.

### 3.3. Effect of 1,3-1,6 β-Glucans Concentration on the Relative Incidence of Muscle Phenotypes (Dystrophic and Non-Dystrophic)

Birefringence is a simple method to assess muscle integrity since the orderly array of muscle fiber generates a signal that is lost upon severe muscle derangement (Figure 1). The birefringence test did not show a significant reduction (*p* ≤ 0.05) in phenotypically dystrophic muscle when the larvae were exposed to 1,3-1,6 β-glucans (Supplementary Figure S1). Despite this, a tendentially higher rate of dystrophic individuals (*p* > 0.05) compared to treated (2, 4 and 8 mg L^−1^) larvae was observed in the untreated (0 mg L^−1^). Overall, based on the above-described results, DNS and NDS treatments were considered as the most appropriate for detecting, testing and observing possible effects of 1,3-1,6 β-glucans on the DMD zebrafish model. Therefore, the effects of 1,3-1,6 β-glucans on larval locomotor performances were tested considering DNS and NDS treatments only.

### 3.4. Locomotor Performances of Dystrophic and Non-Dystrophic Phenotypes (DNS and NDS Procedures)

**Figure 6:**
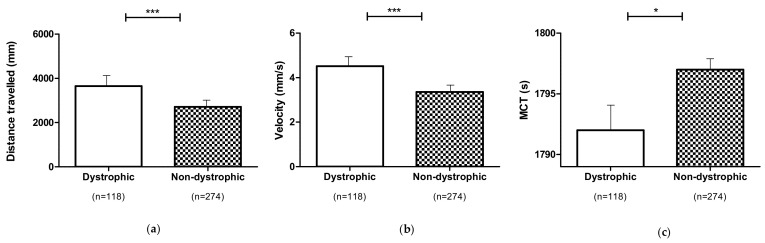
Locomotor performances of dystrophic and non-dystrophic larvae exposed to 1,3-1,6 β-glucans treated (2, 4 and 8 mg L^−1^) according to the DNS and NDS procedure: (**a**) Distance travelled; (**b**) Velocity; (**c**) MCT. Data are given as mean and standard error. (* *p* ≤ 0.05, *** *p* ≤ 0.001).

When embryos were treated at any 1,3-1,6 β-glucans concentration (2, 4 and 8 mg L^−1^) according to the DNS and NDS procedures, locomotor performances of dystrophic larvae were enhanced in comparison to those of non-dystrophic ones. In detail, distance travelled and velocity (*p* ≤ 0.01) increased, while MCT (*p* ≤ 0.05) decreased significantly in comparison to non-dystrophic larvae (Figure 6).

### 3.5. Effect of 1,3-1,6 β-Glucans Concentration on the Relative Incidence of Dystrophic and Non-Dystrophic Phenotypes (DNS and NDS Procedures)

**Figure 7:**
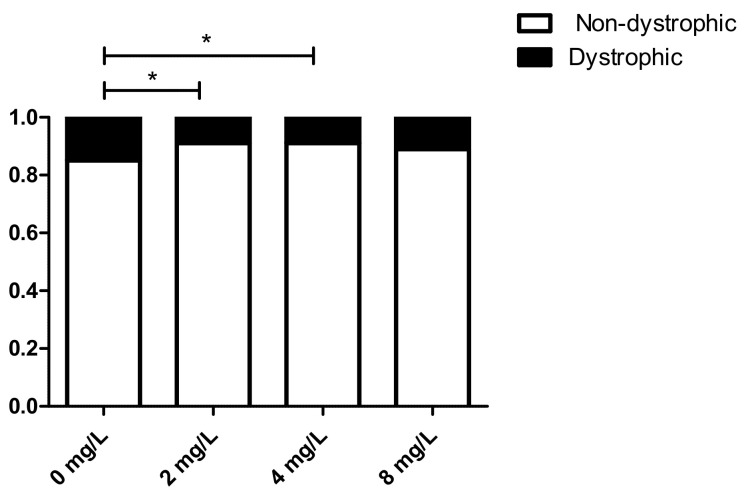
Relative incidence of phenotypes (dystrophic and non-dystrophic) as a function of the concentration of 1,3-1,6 β-glucans in DNS and NDS treatments. (* *p* ≤ 0.05).

The birefringence test of larvae treated according to the DNS and NDS procedures and regardless of the 1,3-1,6 β-glucans treatment concentration showed a significant reduction of phenotypically dystrophic muscle. This analysis revealed significant (*p* ≤ 0.05) decrease of the dystrophic muscle phenotypes with the concentrations of 2 and 4 mg L^−1^ compared to untreated (0 mg L^−1^) subjects (Figure 7). While the reduced incidence of dystrophic phenotypes was not statistically significant when the embryo was exposed to 8 mg L^−1^ 1,3-1,6 β-glucans neither in comparison to the untreated (0 mg L^−1^) group or to the other concentrations used.

### 3.6. Effect of 1,3-1,6 β-Glucans Concentration on Locomotor Performances (DNS and NDS Procedures)

#### 3.6.1. Effect of 1,3-1,6 β-Glucans Concentration on Locomotor Performances Independent of the Individual Phenotypes

Regardless of the individual’s phenotypes, when larvae were treated according to the DNS and NDS procedures, locomotor performances were enhanced for those individuals exposed to 8 mg L^−1^. Larvae travelled a greater distance (*p* ≤ 0.001) at higher velocity (*p* ≤ 0.001). Furthermore, a significant difference was observed among the subjects treated with 2 and 4 mg L^−1^ and the untreated (0 mg L^−1^) larvae. As per MCT, all treated subjects showed similar values, higher than those observed for the untreated (0 mg L^−1^) group (*p* ≤ 0.001; Supplementary Figure S2).

#### 3.6.2. Effect of 1,3-1,6 β-Glucans Concentration on Locomotor Performances According to the Different Phenotypes

**Figure 8:**
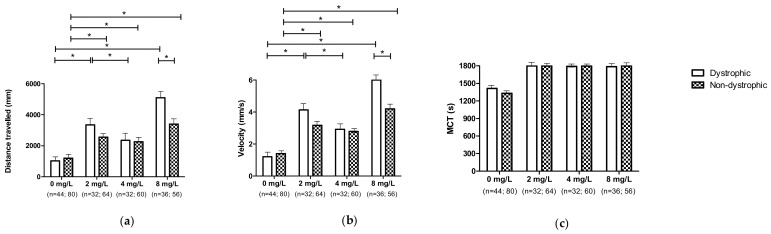
Locomotor performances of DNS and NDS dystrophic and non-dystrophic larvae, untreated (0 mg L^−1^) and treated (2, 4 and 8 mg L^−1^) with 1,3-1,6 β-glucans: (**a**) Distance travelled; (**b**) Velocity; (**c**) MCT. Data are given as mean and standard error (*n* values of each mean are given between brackets). (* *p* ≤ 0.05).

Finally, the treatment with different 1,3-1,6 β-glucans concentration showed a positive effect on locomotor performances. Notably, distance travelled and velocity (*p* ≤ 0.05) of dystrophic larvae exposed to 8 mg L^−1^ was higher compared to untreated (0 mg L^−1^) and larvae exposed to 4 mg L^−1^ (Figure 8). In dystrophic larvae a significant increase (*p* ≤ 0.05) in distance and velocity was observed at the concentration of 2 mg L^−1^ compared to both the untreated (0 mg L^−1^) group and the group treated with 4 mg L^−1^ of 1,3-1,6 β-glucans. At the concentration of 8 mg L^−1^, the distance and velocity of the dystrophic subjects were significantly enhanced in comparison to the untreated (0 mg L^−1^) dystrophic larvae (*p* ≤ 0.05), but not compared to those treated with 2 and 4 mg L^−1^. Moreover, non-dystrophic larvae showed an enhanced distance travelled and velocity when treated with 2 and 8 mg L^−1^ compared to the untreated (0 mg L^−1^) larvae (*p* ≤ 0.05). Conversely, non-dystrophic larvae travelled the same distance and velocity either when treated with 2 or 4 mg L^−1^. Furthermore, treating with 8 mg L^−1^, distance travelled and velocity were significantly enhanced in dystrophic larvae compared to the non-dystrophic (*p* ≤ 0.05). It should be noted that at 0, 2, 4 mg L^−1^ differences were not observed between dystrophic and non-dystrophic subjects. Despite that, a moderate enhancement of dystrophic larvae locomotor performances was observed, even though not significantly (*p* > 0.05). Regarding MCT, no differences were observed according to the 1,3-1,6 β-glucans treatment concentrations in both dystrophic and non-dystrophic larvae, as well as between dystrophic and non-dystrophic larvae within the same concentrations.

### 3.7. Effect of 1,3-1,6 β-Glucans on the Inflammatory Cytokine Tumor Necrosis Factor α (TNF α)

**Figure 9:**
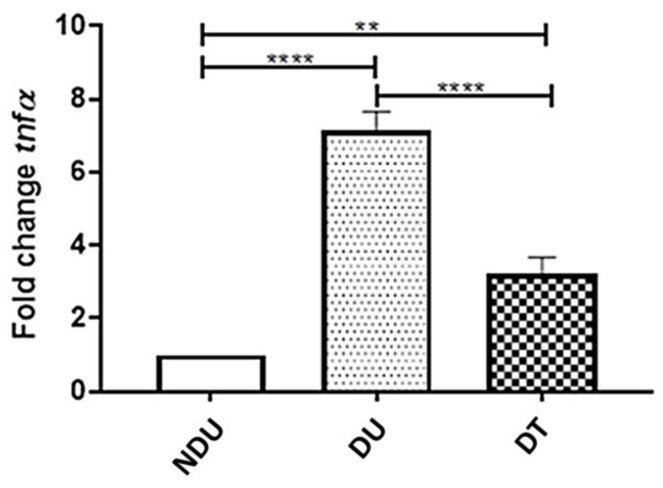
qRT-PCR analysis of DNS and NDS dystrophic and non-dystrophic larvae, untreated (0 mg L^−1^) and treated (2, 4 and 8 mg L^−1^) with 1,3-1,6 β-glucans. Three independent RNA samples from each group were evaluated: Non-dystrophic Untreated (NDU), Dystrophic Untreated (DU), Dystrophic Treated (DT). (** *p* ≤ 0,001, **** *p* ≤ 0.00001).

mRNA expression of the inflammatory cytokine TNF-α was performed by qRT-PCR analysis in untreated (0 mg L^−^^1^) dystrophic and non-dystrophic larvae and dystrophic and non-dystrophic larvae exposed to 8 mg L^−1^ of 1,3-1,6 β-glucans according to the DNS procedure. The results showed a decrease of TNF-α mRNA expression in dystrophic treated (8 mg L^−1^) larvae (Figure 9), suggesting a significant effect of 1,3-1,6 β-glucans on DMD inflammatory state.

### 3.8. Mitochondrial Respiratory Analysis

**Figure 10:**
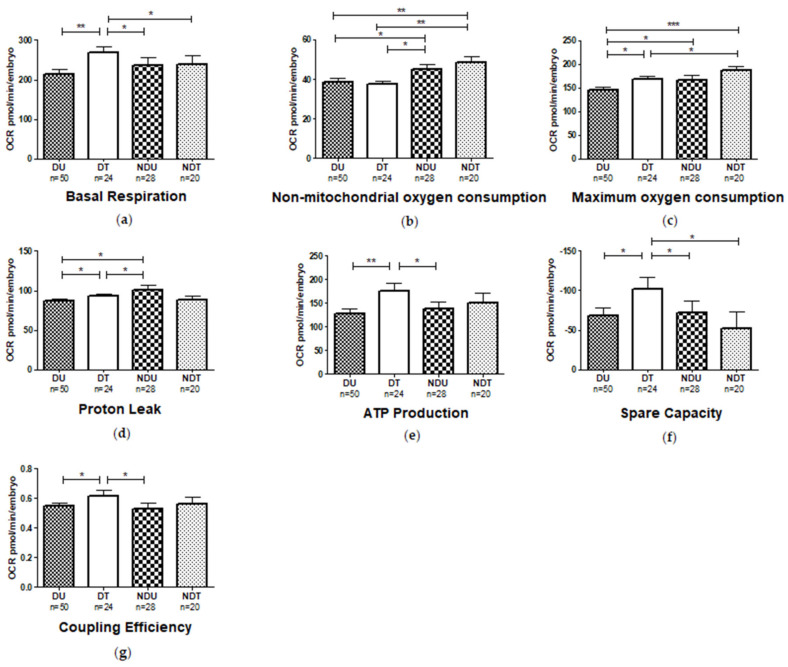
Mitochondrial respiratory analysis of Dystrophic Untreated (DU), Dystrophic Treated (DT), Non-dystrophic Untreated (NDU), Non-dystrophic Treated (NDT): (**a**) Basal respiration; (**b**) Non-mitochondrial oxygen consumption; (**c**) Maximum oxygen consumption; (**d**) Proton Leak; (**e**) ATP Production; (**f**) Spare respiratory capacity; (**g**) Coupling efficiency. Data are given as mean and standard error (* *p* ≤ 0.05, ** *p* ≤ 0.01, *** *p* ≤ 0.001).

Mitochondrial respiratory analysis was performed on dechorionated embryos treated with non-sonicated 1,3-1,6 β-glucans (DNS)**,** exposed to 8 mg L^−1^ and the following parameters were measured: basal respiration, consumption of non-mitochondrial oxygen, ATP production, maximum respiration, proton loss, spare respiratory capacity, coupling efficiency (Supplementary Figure S3). The energy demand of embryos when in basal condition (basal respiration) significantly increased in the dystrophic larvae treated with 1,3-1,6 β-glucans (8 mg L^−1^) compared to the untreated (0 mg L^−1^) dystrophic siblings (*p* ≤ 0.001). In contrast, no differences were observed within non-dystrophic individuals regardless of treatment. In any case, the latter showed a reduced basal respiration compared to the treated (8 mg L^−1^) dystrophic ones (*p* ≤ 0.05; Figure 10a). Regarding the non-mitochondrial oxygen consumption, higher values were observed in the non-dystrophic individuals (untreated and treated) compared to the dystrophic (untreated and treated) larvae (Figure 10b).

Conversely, the maximum oxygen consumption, which shows the maximum rate of respiration that the cell can achieve, was increased in treated (8 mg L^−1^) dystrophic larvae compared to the untreated (0 mg L^−1^) ones (*p* ≤ 0.05). Moreover, untreated (0 mg L^−1^) non-dystrophic larvae showed a higher maximum oxygen consumption compared to dystrophic subjects (Figure 10c). The treatment significantly increased proton leak in treated (8 mg L^−1^) dystrophic larvae, while no differences were observed in wild type larvae (*p* ≤ 0.05). In addition, a lower loss of proton leak was observed in the untreated (0 mg L^−1^) and treated (8 mg L^−1^) dystrophic larvae compared to untreated (0 mg L^−1^) wild types (*p* ≤ 0.05; Figure 10d). Besides, ATP production was significantly increased β-glucans treated (8 mg L^−1^) dystrophic larvae compared to untreated (0 mg L^−1^) ones (*p* ≤ 0.001). Moreover, a difference was observed between treated (8 mg L^−1^) dystrophic larvae and untreated (0 mg L^−1^) non-dystrophic larvae (*p* ≤ 0.05; Figure 10e). On the other side, the spare respiratory capacity was significantly increased in treated (8 mg L^−1^) dystrophic larvae compared to the untreated (0 mg L^−1^) dystrophic ones as well as to the non-dystrophic larvae independently from the treatment (*p* ≤ 0.05; Figure 10f). The coupling efficiency was increased in treated (8 mg L^−1^) dystrophic compared to untreated (0 mg L^−1^) ones (*p* ≤ 0.05), and no difference was observed between treated (8 mg L^−1^) and untreated (0 mg L^−1^) non-dystrophic individuals. Furthermore, the untreated (0 mg L^−1^) dystrophic larvae showed no difference in coupling efficiency compared to non-dystrophic individuals. It is also worthy to be highlighted that in the treated (8 mg L^−1^) dystrophic larvae this value was increased in comparison to the untreated (0 mg L^−1^) non-dystrophic (*p* ≤ 0.05; Figure 10g).

## 4. Discussion

The present study used zebrafish *sapje*, a model of Duchenne muscular dystrophy (DMD) already described by many authors [48,49,50,51,52]. The study evaluated for the first time the effect of 1,3-1,6 β-glucans exposure of zebrafish dystrophic embryos. The assessment of a suitable way for efficiently delivering 1,3-1,6 β-glucans to embryos was also investigated. To this purpose, the effect of embryo dechorionation, β-glucans sonication, and their combination were studied. Indeed, both the presence or absence of the chorion, and the sonication of β-glucans may significantly affect the effectiveness of the treatments [54,55,56,57]. In fact, the efficacy of the tested product (1,3-1,6 β-glucans) may be hindered or limited by the chorionic barrier due to the intrinsic characteristics of the chorion itself or because of the high degree of agglomeration of the tested molecules. This latter might be the case of 1,3-1,6 β-glucans since its high molecular weight and degree of branches may prevent the passage through the chorionic barrier.

As first results, the study confirmed a reduced locomotor performance of dystrophic larvae, in line with the findings of previous studies [48,50]. Second, the effects of different combinations of treatments were evaluated to find out which procedure allows efficient embryos delivery of the molecule. To this regard, it was observed that the enzymatic embryo dechorionation (at 24 hpf) enhanced locomotor performance. Similarly, locomotor performances were also enhanced more efficiently when non-sonicated β-glucans rather than sonicated were used. This result is in contrast with those observed by others [55] in the mouse model since the author reported an increased number of macrophages when sonicated β-glucans were used. This difference could be explained by the more intense sonication performed elsewhere [55], but it does not explain the worsening of the biological activity observed in the present study following β-glucans sonication. A possible alternative hypothesis might be the diverse “endpoint” adopted (percentage of peritoneal macrophages in that study versus locomotor performances in the present study). Other possible explanations may be related to the different route of administration of the β-glucans, as well as to trans-species differences [54]. Furthermore, other authors studied the effect of ultrasonic waves on the structure of β-glucans, showing that they can cause the degradation of the β-glucan molecular chains by breaking the glycosidic links, thus reducing the molecular weight of β-glucan fractions. This modification seems to determine a better β-glucans water solubility and a reduction of the potency of their biological activity [63,64,65]. Based on these observations, it is possible that a correct sonication determines a greater β-glucans solubility and a higher suspension turbidity and viscosity, but it could limit their biological activity. This statement is confirmed by results related to the combined effect of embryo dechorionation and β-glucans sonication (Section 3.2.3), notably for MCT (Figure 5c). Summarizing, the locomotor performances of the larvae were mainly improved with the dechorionation and the use of non-sonicated β-glucans, which resulted to be the most effective method to zebrafish embryos. The reason why non-dechorionated embryos treated with non-sonicated β-glucans showed the worse locomotor performance is probably related to the high size of non-sonicated 1,3-1,6 β-glucans (150–200 kDa), dimensions that impair their passage through the chorion. This hypothesis is supported by the findings reported by other authors [66,67,68], arguing that the size of different substances can represent an impediment to its passage through the chorionic barrier. Thus, as also suggested by the results observed for NDNS and DS treatments, it was decided to use only DNS and NDS approaches for evaluating the efficacy of different concentrations of 1,3-1,6 β-glucans on dystrophic and non-dystrophic zebrafish embryos.

As reported by several authors [48,50] and confirmed by the results observed in the present study, dystrophic larvae showed reduced locomotor performance, travelling less distance at lower velocity compared to non-dystrophic siblings. Upon treatment with 1,3-1,6 β-glucans, dystrophic larvae travelled longer distances at higher velocity than non-dystrophic ones. This result suggests that 1,3-1,6 β-glucans improves locomotor activity and mitochondrial respiration despite the impaired muscular and mitochondrial function typical of muscular dystrophy, by counteracting muscle dysregulations in dystrophic larvae [69]. Similar evidence has been already reported by [49,50,51,52] for compounds different from β-glucans.

Locomotor performances were improved in both dystrophic and non-dystrophic phenotypes when 8 mg L^−1^ 1,3-1,6 β-glucans were used. However, even to a lesser extent, also the treatments at lower concentrations (2 and 4 mg L^−1^) ameliorate the locomotion, this suggesting a dose-dependent effect. In detail, MCT was significantly increased by all concentrations tested and this positive effect was particularly evident in dystrophic *sapje* treated with 8 mg L^−1^**.** In addition, the decreased expression of the inflammatory cytokine TNF-*α* in treated (8 mg L^−1^) dystrophic larvae compared to the untreated (0 mg L^−1^) ones, also showed the anti-inflammatory activity of β-glucans. These results propose a possible use of 1,3-1,6 β-glucans for treating DMD in other models and speculations on its use in DMD patients.

To uncover the underlying mechanisms, mitochondrial function of larvae under 8 mg L^−1^ 1,3-1,6 β-glucan treatment was tested to explore effects on oxidative metabolism. Recent data from other groups [52] have already investigated the possible mitochondrial defect in *sapje* mutants, at least in part. Further, the functional and structural damage of mitochondria in muscle pathologies have already been described in the literature [70,71,72,73]. Sarcolemma defects typical of DMD, cause increased Ca^2+^ inflow, which in turn cause a functional and structural alteration of the mitochondria such as ATP depletion [74]. The function of the mitophagy is essential to restore mitochondrial quality and number, thus limiting cellular degeneration. In particular, the action of β-glucans determines an increase in phagocytosis, which can lead to an increase in autophagy and thus in mitophagy. Therefore β-glucans can play an action in mitophagy and as antioxidants [44,45,46,47] and protect against mitochondrial dysfunction [43]. Unlike previous data [52], it was observed that the basal respiration of untreated (0 mg L^−1^) dystrophic subjects is similar to that of healthy larvae. The possible discrepancy between these studies may be due to a different developmental stage of the embryos. Indeed, in that study 72 hpf larvae instead of 120 hpf larvae were used. Nonetheless, significant differences in the other parameters of mitochondrial respiration were observed between the two phenotypes. In particular, reduced proton leak, maximal respiration and non-mitochondrial oxygen consumption indicated mitochondrial damage in dystrophic larvae compared to non-dystrophic subjects. It is worthwhile to recall that proton leak represents the remaining basal respiration not coupled to ATP production. The lower values of proton leak observed in dystrophic larvae do not necessarily mean mitochondrial damage. However, considering the high levels of cholesterol that occurs in muscle tissue of DMD patients [75], which perturbs mitochondrial membrane physical properties and morphology and disrupts the assembly of mitochondrial respiratory super-complexes [76], this cholesterol enrichment may be responsible for a lower passive proton permeability, and hence a lower proton leak [77]. The slight increase of proton leak observed, after β-glucans administration in dystrophic larvae, was probably caused by the increased basal respiration. It is also possible to argue that this faint effect could be caused by the cholesterol-lowering effect of β-glucans [78]. The non-mitochondrial oxygen consumption is the oxygen consumed by processes outside the mitochondria, important to get an accuracy measure of mitochondrial respiration. The non-mitochondrial oxygen-consuming processes are not well defined, but they are predominantly those that originate from pro-oxidant and pro-inflammatory enzymes and are regarded as negative indicators of bioenergetic health. Therefore, a reduction of non-mitochondrial oxygen consumption implied an overall slowing down of cell metabolism due to energy stress.

This confirms the mitochondrial dysregulation of the dystrophic larvae. Further, the results of the present study showed that the treatment with 1,3-1,6 β-glucans causes beneficial effects on mitochondrial function of the dystrophic subjects, through the significant improvement of basal respiration, maximal respiration, ATP production, spare respiratory capacity, and coupling efficiency. However, neither a decrease of the ATP production nor of the coupling efficiency in dystrophic larvae was observed compared to non-dystrophic ones, as already described in [79,80]. This is probably due to the early developmental stage analysed, since the mitochondrial dysfunction in DMD is not direct, the metabolic defects may appear later in development when the mitochondria are unable to counterbalance cell dysfunction. Indeed, a reduced maximum oxygen consumption was observed in dystrophic larvae, that was estimated by the FCCP-stimulated respiration, and when it is reduced, is a strong indicator of potential mitochondrial dysfunction. Furthermore, its increment after 1,3-1,6 β-glucans treatment showed to rescue the damage. Thereafter, the improvement of the key parameters of mitochondrial function by directly measuring the oxygen consumption rate appears critical to sustain muscular function and are possibly linked to improved velocity and locomotion in treated (8 mg L^−1^) larvae. Results are in line with what observed in [52], showing a significant improvement in mitochondrial respiration by observing only basal respiration using Alisporivir drug [52]. As a final comment, the present study also showed the effect of 1,3-1,6 β-glucans on wild type zebrafish, displaying an increased mitochondrial functionality compared to untreated (0 mg L^−1^) ones. Considering that the complexes III and IV of the mitochondrial respiratory chain in DMD are impaired promoting mitochondrial dysfunction, and eventually leading to progressive weakness and muscle wasting [66], the improvement of mitochondrial respiration obtained under 1,3-1,6 β-glucans administration, could contribute to increase muscle strength, which induce to an increase of the locomotion in *sapje* treated (8 mg L^−1^) larvae, indicating the efficacy of treatment. These findings suggested that 1,3-1,6 β-glucans protect mitochondria in normal conditions, but exert their effect mostly when they are damaged, as in the case of dystrophic larvae. Indeed, it should be noted that the treatment with 1,3-1,6 β-glucans increased basal respiration in dystrophic larvae while no effect was observed in non-dystrophic larvae. Moreover, all the other parameters of OCR increased in treated (8 mg L^−1^) dystrophic larvae. Since basal respiration is determined by the production of ATP and proton leak, these results suggest that 1,3-1,6 β-glucans act on impaired basal respiration through ATP production and proton leak enhancement. These results are in line with what was found in [74], showing beneficial effects of 1,3-1,6 β-glucans on mitochondria with severe dysfunctions.

## 5. Conclusions

The main goal of the present study was to investigate whether the administration of 1,3-1,6 β-glucans to DMD patients may positively affect their well-being. To this purpose, *sapje* embryos were used as an animal model of DMD since it recapitulates the key features of the human disease.

The first step of the study was to find out the most suitable methods of β-glucans administration to avoid possible biased results (false negative mainly). In this regard, it was observed that, only when combining embryo dechorionation with the use of non-sonicated β-glucans (DNS), and sonicated β-glucans with non-dechorionated embryos (NDS), were 1,3-1,6 β-glucans exerting positive effects.

Afterwards, in a dose–effect relationship, 8 mg L^−1^ 1,3-1,6 β-glucans enhanced locomotor performances while a significant reduction of the incidence of dystrophic phenotypes was achieved with lower concentrations. Finally, these results were consistent with the amelioration observed on DMD inflammatory state (TNF-α mRNA expression) and the mitochondrial function of dystrophic larvae (mitochondrial respiration).

Despite these promising results, suggesting 1,3-1,6 β-glucans as a valid food supplement in the diets for DMD patients, further studies are needed for uncovering the pathway by which they regulate locomotion and mitochondrial function.

## Data Availability

Additional data related to this study are available on request from the corresponding author.

## Supplementary Materials

The following are available online at https://www.mdpi.com/article/10.3390/nu13051619/s1, Figure S1: Relative incidence of phenotypes (dystrophic and non-dystrophic) as a function of the concentration of β-glucans, Figure S2: Locomotor performances of larvae treated according to the DNS and NDS procedure and exposed to 0, 2, 4 and 8 mg L-1 of 1,3-1,6 β-glucans: (a) Distance travelled; (b) Velocity; (c) MCT. Data are given as mean and standard error (ns *p* > 0.05, *** *p* ≤ 0.001), Figure S3: An example of the OCR line graph that is generated with the different stages of the mitochondrial respiration protocol, Table S1. List and sequences of the qRT-PCR primers used.
