# Supplementary material for: β-Glucans as Dietary Supplement to Improve Locomotion and Mitochondrial Respiration in a Model of Duchenne Muscular Dystrophy
Source: Nutrients. 2021 May 12;13(5):1619. doi: 10.3390/nu13051619 (PMC8151547; doi:10.3390/nu13051619)
Supplement: Supplementary file 1 [file nutrients-13-01619-s001.zip › nutrients-1160555-supplementary.pdf]

Supplementary Material

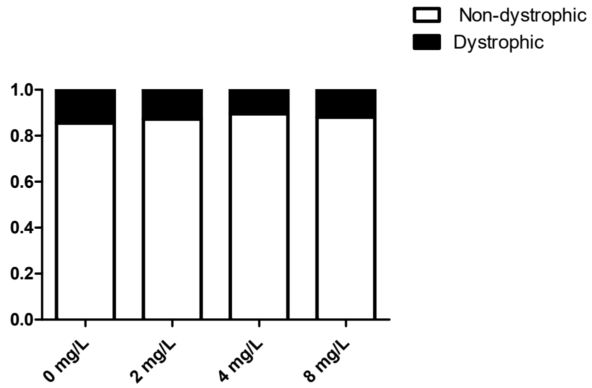

Figure S1. Relative incidence of phenotypes (dystrophic and non-dystrophic) as a function of the concentration of β-glucans

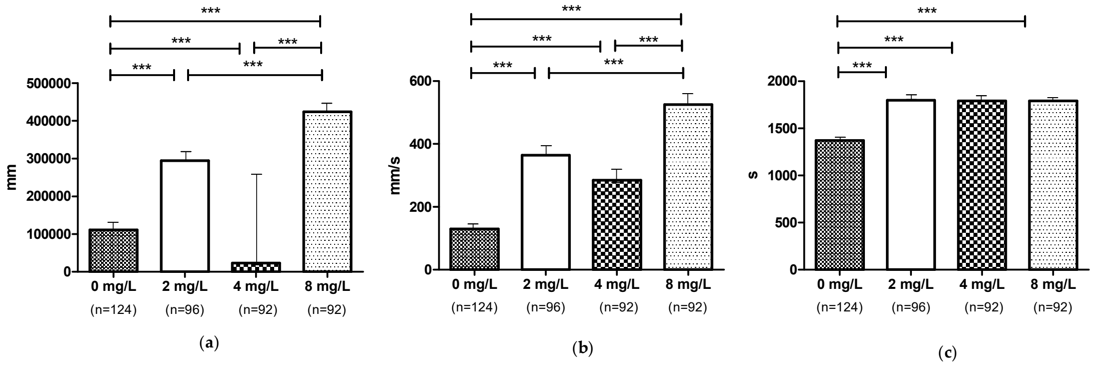

Figure S2. Locomotor performances of larvae treated according to the DNS and NDS procedure and exposed to 0, 2, 4 and 8 mg L-1 of 1,3-1,6 β-glucans: (**a**) Distance travelled; (**b**) Velocity; (**c**) MCT. Data are given as mean and standard error (ns P > 0.05, *** P ≤ 0.001)

Table S1. List and sequences of the qRT-PCR primers used.

B-ACT Fw	5’-GCAGAAGGAGATCACATCCCTGGC-3’
B-ACT Rv	5’-CATTGCCGTCACCTTCACCGTTC-3’
Tnfa-Fw	5’-TCGGGTGTATGGAGGGTGTT-3’
Tnfa-Rv	5’-CTGGGTCTTATGGAGCGTGA-3’

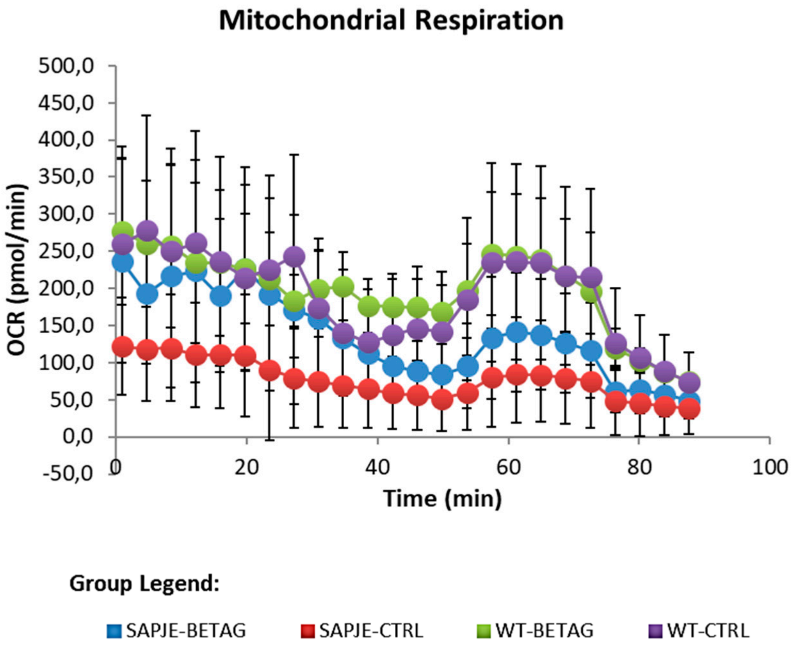

Figure S3. An example of the OCR line graph that is generated with the different stages of the mitochondrial respiration protocol
